# The impact of age, sex, cardio-respiratory fitness, and cardiovascular disease risk on dynamic cerebral autoregulation and baroreflex sensitivity

**DOI:** 10.1007/s00421-022-04933-3

**Published:** 2022-04-16

**Authors:** Joseph D. Maxwell, Daniel J. Bannell, Aine Brislane, Sophie E. Carter, Gemma D. Miller, Kirsty A. Roberts, Nicola D. Hopkins, David A. Low, Howard H. Carter, Andrew Thompson, Jurgen A. H. R. Claassen, Dick H. J. Thijssen, Helen Jones

**Affiliations:** 1 Research Institute of Sport and Exercise Science, Liverpool John Moores University, Tom Reilly Building, Byrom Street, Liverpool, L3 3AF UK; 2 School of Sport, York St. John University, York, UK; 3 School of Sport Science, Exercise and Health, The University of Western Australia, Perth, Australia; 4 Wolfson Centre for Personalised Medicine, Institute of Translational Medicine, University of Liverpool, Liverpool, UK; 5 Department of Geriatric Medicine and Donders Institute for Brain, Cognition and Behaviour, Radboud University Medical Center, Nijmegen, The Netherlands; 6 Department of Physiology, Radboud Institute of Health Sciences, Radboud University Medical Center, Nijmegen, The Netherlands

**Keywords:** Cerebral autoregulation, Cardiac baroreflex sensitivity, Cardio-respiratory fitness, Ageing

## Abstract

**Background:**

Humans display an age-related decline in cerebral blood flow and increase in blood pressure (BP), but changes in the underlying control mechanisms across the lifespan are less well understood. We aimed to; (1) examine the impact of age, sex, cardiovascular disease (CVD) risk, and cardio-respiratory fitness on dynamic cerebral autoregulation and cardiac baroreflex sensitivity, and (2) explore the relationships between dynamic cerebral autoregulation (dCA) and cardiac baroreflex sensitivity (cBRS).

**Methods:**

206 participants aged 18–70 years were stratified into age categories. Cerebral blood flow velocity was measured using transcranial Doppler ultrasound. Repeated squat-stand manoeuvres were performed (0.10 Hz), and transfer function analysis was used to assess dCA and cBRS. Multivariable linear regression was used to examine the influence of age, sex, CVD risk, and cardio-respiratory fitness on dCA and cBRS. Linear models determined the relationship between dCA and cBRS.

**Results:**

Age, sex, CVD risk, and cardio-respiratory fitness did not impact dCA normalised gain, phase, or coherence with minimal change in all models (*P* > 0.05). cBRS gain was attenuated with age when adjusted for sex and CVD risk (young–older; *β* = − 2.86 *P* < 0.001) along with cBRS phase (young–older; *β* = − 0.44, *P* < 0.001). There was no correlation between dCA normalised gain and phase with either parameter of cBRS.

**Conclusion:**

Ageing was associated with a decreased cBRS, but dCA appears to remain unchanged. Additionally, our data suggest that sex, CVD risk, and cardio-respiratory fitness have little effect.

## Introduction

Ageing is a non-modifiable risk factor for cerebrovascular diseases (Boehme et al. 2017). Evidence shows that both cerebral blood flow (CBF) (Lu et al. 2011) and cerebral blood flow velocity (CBFv) decline with age (Ainslie et al. 2008). Yet, age-related changes in cerebrovascular function and its interaction with systemic haemodynamic regulation are not well established. Within the cerebrovasculature, the intrinsic ability to maintain adequate CBF in the presence of transient changes in blood pressure (BP) that occur over a number of seconds is referred to as dynamic cerebral autoregulation (dCA) (Aaslid et al. 1989; Claassen et al. 2016). dCA acts as a defensive mechanism protecting the brain from potential damage from high or low BP (van Beek et al. 2008). Simultaneously neural control of systemic BP occurs via the baroreceptors, yet the relationship between these two regulating processes has not been well studied and may provide insightful mechanistic information into CBF regulation. Indeed, whether changes in BP control alter acute cerebral haemodynamics may in turn provide a potential target for interventions.

Previous research assessing dCA using forced BP oscillations with repeated squat-stand manoeuvres has shown, despite age-related reductions in CBFv and increases in BP (Ainslie et al. 2008), there is little evidence of impairment in dCA between groups of young and old (mean age 23 vs 66 years) healthy individuals (Smirl et al. 2015). A finding which has also been replicated within clinical populations (e.g., Alzheimer’s) (Claassen et al. 2009a; Smirl et al. 2014; Lewis et al. 2019). Xing et al. (2017), in a larger sub-sample of individuals across the age range 18–70 years, also observed that dCA from driven oscillations was not different across the lifespan in healthy individuals free of cardiovascular disease (CVD). Although there was some evidence that women had a better dCA compared to men, this finding was in contrast to a recent study (Labrecque et al. 2019a). One research group have also highlighted the potential importance of cardio-respiratory fitness when assessing sex differences in dCA (Labrecque et al. 2017, 2019a, b). Despite this, no study has examined the interaction of sex, cardio-respiratory fitness, or CVD risk factors on dCA in a large sample of individuals across the life span.

Ageing negatively influences cardiac BRS (cBRS) (Monahan 2007), with conflicting evidence as to whether there are sex differences in this response (Xing et al. 2017; Okada et al. 2012), and Hart et al. (2011). cBRS positively correlates with dCA in young but not middle aged or older healthy participants (Xing et al. 2017). However, the fundamental relationship between dCA and cBRS is unclear as other studies suggest an inverse relationship in young healthy individuals (Tzeng et al. 2010), and no relationship in older endurance trained athletes (Aengevaeren et al. 2013) or in heart transplant recipients (Smirl et al. 2014). Understanding such relationships is further complicated by the use of a number of different techniques to bring about changes in BP and analysis methods to quantify dCA and cBRS. Our aim was twofold; (1) to examine the impact of sex, cardio-respiratory fitness, and CVD risk factors on dCA and cBRS over the life span; and (2) to explore the relationships between cBRS and dCA whilst controlling for age and sex. To address these aims, we used secondary data from studies undertaken in our laboratory, that employed the same technique to bring about changes in BP (repeated squat-stand manoeuvres) and analysis method (transfer function analysis) (Claassen et al. 2016), in a large sample of individuals.

## Methods

### Participants

Data from 11 studies collected at Liverpool John Moores University, Research Institute for Sport and Exercise Science were examined for eligibility. Data were included if: (1) all measurements were performed with strict adherence to Cerebral Autoregulation Network (CARNet) guidelines (Claassen et al. 2016), (2) individual-level minimum dataset was available [i.e., age, sex, body mass index (BMI), and resting BP], and (3) data were collected in studies that adhered to the Declaration of Helsinki. Data were included from four previously published studies (Carter et al. 2018, 2020; Maxwell et al. 2019; Brislane et al. 2020) where dCA and cBRS recordings were collected with corresponding participant characteristics and medical history (where available). When studies adopted a repeated-measures design, only baseline data were included. Participant data was excluded if the duration of recordings was < 5-min, and if the coherence value was < 0.4 (Claassen et al. 2016). Based on these criteria, 206 participants were included consisting 83 males and 123 females aged between 18 and 70 years. All participants were non-smokers, with no previous myocardial infarction, stroke, or thrombosis. Individuals clinically diagnosed with Type 2 diabetes mellitus (T2DM) were treated with Metformin (*n* = 18) or diet (*n* = 8) at the time of data collection. Additional medications taken by participants included anti-hypertensive (*n* = 15) and lipid lowering (*n* = 16) medication. Participants that had a BMI > 30 kg/m^2^, diagnosed with hypercholesterolemia or T2DM, as well treated or untreated ≥ stage 1 hypertension were stratified to a CVD risk group. Fifty eight of the females were post-menopausal. These women were classified based on having no menstrual cycle for at least 12 consecutive months and not previously or currently taking any form of hormone therapy (Moreau et al. 2012).

### Protocol

All participants arrived at the laboratory following an overnight fast and had refrained from alcohol and exercise for ≥ 24 h, and caffeine for ≥ 12 h, prior to the visit. Following a minimum of 20 min supine rest, measurements of middle cerebral artery velocity (MCAv) were obtained using transcranial Doppler ultrasound (TCD) following standardised procedures (Willie et al. 2011). Two 2-MHz Doppler probes (Spencer Technologies, Seattle, USA) were placed over the temporal window and adjusted until an optimal signal was identified and held in place using a Marc 600 head frame (Spencer Technologies, Seattle, USA). Beat-to-beat blood pressure was recorded using a Finometer (Model 1, Finapres Medical Systems BV, Amsterdam, The Netherlands). Participants were fitted with a photoplethysmographic cuff on the right index finger, and the output was corrected by referencing the cuff to heart level using a height correction unit and heart rate (HR) acquired from a 3-lead electrocardiogram. Partial pressure of end tidal carbon dioxide (P_ET_CO_2_) was continuously monitored by instrumenting participants with a two-way valve mouthpiece (Hans Rudolph) connected to a calibrated gas analyser (ML206 ADinstruments, Colorado Springs, USA). All data were sampled at 50 Hz with the data acquisition system PowerLab via the interface LabChart 7 (ADinstruments, Colorado Springs, USA).

### Baseline haemodynamics

Resting MCAv, HR, mean arterial pressure (MAP) and P_ET_CO_2_ were continuously recorded for 5 min in the supine position. Participants were instructed to maintain normal breathing and refrain from closing their eyes. Baseline data were averaged over the 5-min recording.

### Dynamic cerebral autoregulation

dCA was assessed using repeated squat-stand manoeuvres to induce oscillations in MAP. This technique has been shown to be the best protocol for eliciting high interpretable linearity between MAP and MCAv signals (Smirl et al. 2015; Claassen et al. 2009b). Beginning in a standing position, the participants mimic the experimenter by squatting down obtaining a ≈ 90° angle and then returning to the standing position. The manoeuvres were performed at a frequency of 0.10 Hz (5-s squat–5-s stand) for a period of 5 min. This frequency of manoeuvre was performed as these large oscillations in MAP are extensively buffered by cerebral vessels when completed at frequencies within the high-pass filter buffering range (< 0.20 Hz) (Zhang et al. 1998). By executing repeated squat-stand manoeuvres, this optimises the signal-to-noise ratio and improves the interpretability of the recordings through the physiologically relevant change in MAP (Smirl et al. 2015). Whilst performing the manoeuvres, participants were instructed to maintain normal breathing and avoid Valsalva manoeuvres. Throughout the 5-min protocol, MCAv, HR, MAP, and P_ET_CO_2_ were continuously assessed.

Data for dCA were analysed in accordance with the most recent recommendations from the CARNet (Claassen et al. 2016). Both beat-to-beat MCAv and MAP signals were extracted from LabChart and then spline interpolated before being re-sampled at 4 Hz for spectral analysis and transfer function analysis (TFA) based on the Welch algorithm. Each of the 5-min recordings was subdivided into 5 successive windows overlapping by 50%. Each window was passed through a Hanning window prior to Fourier transformation. The cross spectrum between MCAv and MAP was determined for TFA by the MAP auto-spectrum to determine transfer function parameters absolute gain, normalised gain, phase, and coherence. dCA data (squat–stand manoeuvres) were sampled at the point estimate of the driven frequency (0.10 Hz). TFA parameters were only included for subsequent analysis when coherence exceeded 0.4. Additionally, data were excluded if 5 min of clear artifact free recordings were not present.

### Cardiac baroreflex sensitivity

During the same 5-min 0.10 Hz squats-stand manoeuvres, continuous cBRS was measured. The cBRS was determined by applying TFA to systolic BP and R–R interval (pressure-cardiac interval) at the point estimate of the driven frequency of the squat-stand manoeuvres (0.10 Hz). Data analysis was performed using a commercially available software Ensemble (Version 1.0.0.28, Elucimed, Wellington, New Zealand). Mean gain, phase, and coherence along with spectral power of systolic BP and R–R interval were calculated in the low-frequency range.

### Cardio-respiratory fitness

Breath-by-breath expired gases were continuously monitored (Oxycon Pro, Jaeger, Hochberg Germany) for oxygen consumption (ml/kg^/^min) during an incremental maximal exercise test and were averaged over 15 s (Sprung et al. 2013). Peak oxygen uptake was calculated from the highest consecutive 15-s period of expired gas fractions. All participants reached the criteria for volitional exhaustion based upon heart rate, peak oxygen uptake, Borg scale, and respiratory exchange ratio (Sprung et al. 2013; Bailey et al. 2016).

### Statistical analysis

Statistical analysis was performed using IBM SPSS version 26 (SPSS Inc., Chicago, IL). First, participants were stratified into three age categories: young (18–35 years, *n* = 93), middle age (36–55 years, *n* = 62), and old age (56–70 years, *n* = 51). Between age-category differences in baseline characteristics and power spectrum densities during squat-stand manoeuvres were explored using one-way ANOVA. To examine the influence of age, sex, CVD risk, and *V*O_2max_ linear regression was employed. Cross-sectional associations between age and measures of dCA and cBRS were examined using linear regression adjusting for sex (Model 1). Multivariable linear regression was used to further adjust for health status (model 2) as well as *V*O_2max_ (model 3). To examine specifically changes associated with cBRS and menopause, pre vs post-menopausal women were compared using a general linear model with age as covariate.

#### Relationship between cardiac BRS and dCA

The linear relationship between cBRS and dCA was determined using the Coefficient of determination (*R*^2^). For the models, each parameter of cBRS was independently used as a predictor variable and each parameter of dCA an outcome variable with adjustments for age and sex. Evidence of multicollinearity was explored using the variance inflation factor. Statistical significance was set a *P* < 0.05.

## Results

### Participant characteristics

There was an increase in SBP, DBP, and BMI (*P* < 0.001) and decrease in MCAv and *V*O_2max_ (*P* < 0.001) with age (Table 1) at baseline. Age, SBP, DBP, and BMI were significantly higher (*P* < 0.001) in the CVD risk group compared to healthy, with *V*O_2max_ and MCAv significantly lower in the CVD risk group (*P* < 0.001) (Table 1).

**Table 1:** Participant characteristics when divided into age categories

Characteristics	Age categories			ANOVA
	18–35 years (young) *N* = 93	36–55 years (middle age) *N* = 62	56–70 years (old age) *N* = 51	*P* value
Age (years)	26 ± 5	47 ± 6	61 ± 4	
Male/female	45/48	18/44	20/31	
SBP (mmHg)	115 ± 11	120 ± 15	138 ± 18	< 0.001
DBP (mmHg)	67 ± 11	73 ± 10	78 ± 10	< 0.001
*V*O_2max_ (ml kg min)	42.2 ± 10.8	28.6 ± 7.4	23.7 ± 5.2	< 0.001
BMI (kg/m^2^)	24 ± 3	27 ± 6	29 ± 5	< 0.001
MCAv (cm s)	67 ± 13	64 ± 13	56 ± 13	< 0.001
P_ET_CO_2_ (mmHg)	36.8 ± 4.3	37.9 ± 4.8	35.9 ± 4.9	0.08

	Health status		
	Healthy *N* = 166	CVD Risk *N* = 40	*P* value
Age (years)	37 ± 14	56 ± 3	< 0.001
Male/female	57/109	26/14	
SBP (mmHg)	117 ± 13	145 ± 15	< 0.001
DBP (mmHg)	68 ± 8	83 ± 9	< 0.001
*V*O_2max_ (ml kg min)	34.0 ± 12.0	22.5 ± 5.1	< 0.001
BMI (kg/m^2^)	24.6 ± 3.6	33 ± 5	< 0.001
MCAv (cm s)	65.8 ± 13.4	54 ± 8	< 0.001
P_ET_CO_2_ (mmHg)	36.8 ± 4.4	37.6 ± 5.7	0.37
Medications			
Anti-hypertensive mediation	0/166	19/40	
Metformin	0/166	17/40	
Lipid lowering medication	0/166	16/40	

Data presented as mean ± SD

*SBP* systolic blood pressure, *DBP* diastolic blood pressure, *BMI* body mass index, *MCAv* middle cerebral artery velocity, *PETCO*_*2*_ partial pressure of end tidal carbon dioxide, *ANOVA* analysis of variance

### dCA

Age, sex, CVD risk factors, and *V*O_2max_ did not impact dCA parameters normalised gain, phase or coherence with minimal change (*β*) compared to the young aged reference group (18–35 years) in all statistical models (*P* > 0.05, Table 2). There was a significant reduction in dCA gain with age, which was apparent when adjusted for sex and CVD risk factors (young—middle age; *β* = − 0.09, *P* = 0.02 and young—old age; *β* = − 0.18, *P* < 0.001, model 2) but not when adjusted for *V*O_2max_ (model 3).

**Table 2:** Cross-sectional associations between age and both dCA and cardiac BRS during 0.10 Hz squat-stand manoeuvres

	Mean ± SD	Model 1		Model 2		Model 3	
		*β* (95% CI)	*P* value	*β* (95% CI)	*P* value	*β* (95% CI)	*P* value
dCA normalised gain (%·mmHg^−1^) (years)							
18–35	1.34 ± 0.28	Ref		Ref		Ref	
36–55	1.31 ± 0.30	− 0.04 (− 0.14, 0.06)	0.42	− 0.03 (− 0.14, 0.07)	0.55	0.00 (− 0.18, 0.18)	0.96
56–70	1.29 ± 0.34	− 0.06 (− 0.16, 0.05)	0.29	− 0.04 (− 0.16, 0.08)	0.55	0.01 (− 0.20, 0.21)	0.96
dCA gain (cm/s/mmHg) (years)							
18–35	0.89 ± 0.23	Ref		Ref		Ref	
36–55	0.82 ± 0.22	− 0.09 (− 0.16, − 0.02)	0.01	− 0.09 (− 0.16, − 0.01)	0.02	− 0.05 (− 0.17, 0.08)	0.45
56–70	0.70 ± 0.18	− 0.20 (− 0.28, − 0.13)	< 0.001	− 0.18 (− 0.27, − 0.10)	< 0.001	− 0.22 (− 0.36, − 0.08)	0.002
dCA phase (radians) (years)							
18–35	0.39 ± 0.28	Ref		Ref		Ref	
36–55	0.35 ± 0.32	− 0.007 (− 0.10, 0.08)	0.88	− 0.001 (− 0.09, 0.09)	0.98	− 0.004 (− 0.18, 0.17)	0.96
56–70	0.39 ± 0.24	0.01 (− 0.08, 0.11)	0.78	0.02 (− 0.09, 0.14)	0.67	0.10 (− 0.09, 0.29)	0.29
dCA coherence (years)							
18–35	0.67 ± 0.1	Ref		Ref		Ref	
36–55	0.65 ± 0.1	− 0.02 (− 0.05, 0.02)	0.34	− 0.02 (− 0.06, 0.01)	0.20	− 0.02 (− 0.07, 0.04)	0.55
56–70	0.70 ± 0.1	0.03 (− 0.01, 0.06)	0.18	0.01 (− 0.03, 0.05)	0.59	0.02 (− 0.05, 0.08)	0.58
BRS gain (ms mmHg) (years)							
18–35	5.99 ± 2.96	Ref		Ref		Ref	
36–55	3.57 ± 2.27	− 2.18 (− 3.00, − 1.36)	< 0.001	− 1.85 (− 2.70, − 0.99)	< 0.001	− 0.54 (− 1.67, 0.58)	0.34
56–70	3.01 ± 2.06	− 2.86 (− 3.72, − 1.99)	< 0.001	− 2.21 (− 3.20, − 1.22)	< 0.001	− 0.60 (− 1.87, 0.67)	0.35
BRS phase (radians) (years)							
18–35	− 0.78 ± 0.42	Ref		Ref		Ref	
36–55	− 1.11 ± 0.56	− 0.31 (− 0.48, − 0.14)	< 0.001	− 0.31 (− 0.49, − 0.13)	0.001	− 0.20 (− 0.50, 0.09)	0.18
56–70	− 1.22 ± 0.61	− 0.44 (− 0.60, − 0.25)	< 0.001	− 0.43 (− 0.63, − 0.22)	< 0.001	− 0.28 (− 0.61, 0.06)	0.10
BRS coherence (years)							
18–35	0.70 ± 0.13	Ref		Ref		Ref	
36–55	0.64 ± 0.11	− 0.06 (− 0.09, − 0.02)	0.004	− 0.06 (− 0.10, − 0.02)	0.003	− 0.05 (− 0.11, 0.14)	0.13
56–70	0.67 ± 0.11	− 0.03 (− 0.07, 0.01)	0.13	− 0.04 (− 0.09, − 0.01)	0.11	0.02 (− 0.05, 0.09)	0.60

The regression coefficient β represents the change in the parameter from either young (18–35 years)–middle (36–55 years) aged or from young–old (56–70 yrs) aged when accounting for model covariates. Model 1: Adjusted for sex. Model 2: Adjusted for sex and health status (healthy or CVD risk). Model 3: Adjusted for sex, health status and *V*O_2max_

*dCA* dynamic cerebral autoregulation, *BRS* baroreflex sensitivity

### BRS

cBRS gain was attenuated with age when adjusted for sex and CVD risk factors (young—middle age; *β* = − 2.18, *P* < 0.001 and young—old age; *β* = − 2.86 *P* < 0.001, model 2) along with BRS phase (young—middle age; *β* = − 0.31, *P* < 0.001 and young—old age; *β* = − 0.44 *P* < 0.001, model 2) but not adjusted for *V*O_2max_ (model 3). cBRS gain was significantly lower in the post-menopausal group compared to pre-menopausal (− 1.59 ms/mmHg; 95% CI − 2.43–0.77 *P* < 0.001) but not when using age as a covariate (− 0.50 ms/mmHg; 95% CI − 2.06, 1.06 *P* = 0.79). Similarly, cBRS phase was attenuated in the post-menopausal group compared to pre-menopausal (− 0.39 radians; 95% CI − 0.62, 0.15 *P* = 0.02) but not when using age as a covariate (0.06 radians; 95% CI − 0.36, 0.45 *P* = 0.77).

### Power spectral analysis

When stratified by age, dCA BP power, MCAv power, and cardiac BRS R–R interval power all demonstrated a negative relationship (*P* < 0.001) with no difference in SBP power (*P* = 0.55, Table 3).

**Table 3:** Power spectral analysis of both dynamic cerebral autoregulation and baroreflex sensitivity during 0.10 Hz squat-stand manoeuvres

	Age categories			ANOVA
	18–35 years (young) *N* = 93	36–55 years (middle age) *N* = 62	56–70 years (old age) *N* = 51	
dCA				
BP power (mmHg^2^)	215 ± 128	172 ± 105	140 ± 124	0.001
MCAv power (cm/s^2^)	166 ± 97	132 ± 91	60 ± 41	< 0.001
BRS				
R–R interval power (ms^2^)	8916 ± 6932	4390 ± 5190	3146 ± 3991	< 0.001
SBP power (mmHg^2^)	474 ± 368	412 ± 299	470 ± 429	0.55

Values are mean ± SD

*BP* blood pressure, *BRS* baroreflex sensitivity, *dCA* dynamic cerebral autoregulation, *MCAv* middle cerebral artery velocity, *SBP* systolic blood pressure

### Relationship between cBRS and dCA

**Fig. 1:**
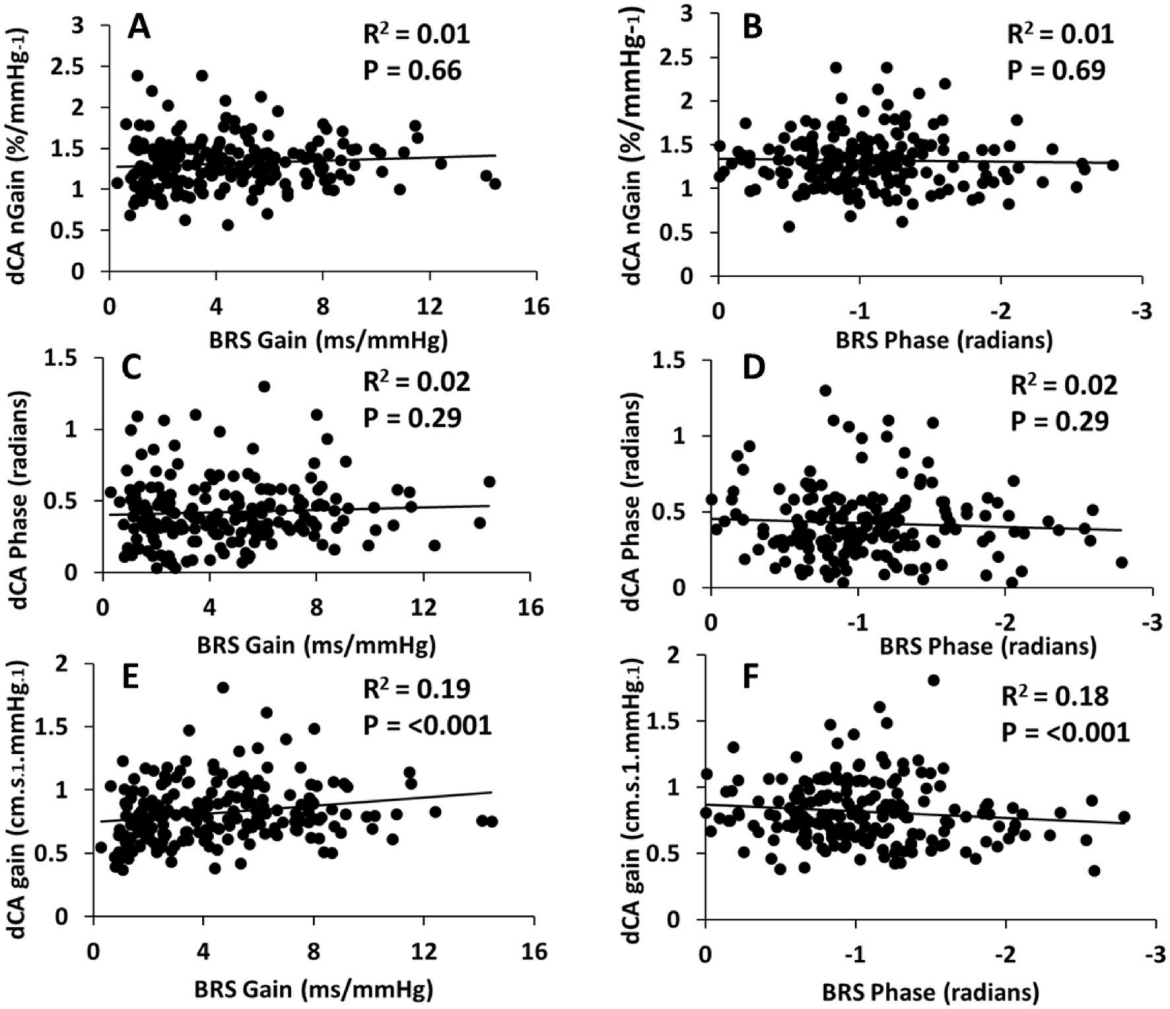
Relationship between dynamic cerebral autoregulation and baroreflex sensitivity during 0.10 Hz squat-stand manoeuvres. Data presented as individual data points with *R*^2^ and *P* values

There was little correlation between dCA normalised gain and dCA phase with either parameter of cBRS (*P* > 0.05; Fig. 1). dCA gain was correlated with cBRS gain (*R*^2^ = 0.19, *P* < 0.001) and with cBRS phase (*R*^2^ = 0.18, *P* < 0.001). However, the total variance explained in these significant outcomes is small, meaning that other factors are likely to be important, whether independent or as interacting variables.

## Discussion

The aims of the current study were to (1) examine the impact of sex, cardio-respiratory fitness and the presence of CVD risk factors on dCA and cBRS over the life span; and (2) explore the relationships between cBRS and dCA whilst controlling for age and sex. We present the following observations. First, dCA measured using repeated squat-stand manoeuvres is preserved across the age range of 18–70 years in healthy individuals ex, fitness or the presence of CVD risk factors had little effect. Second, cBRS declined with ageing. Finally, cBRS gain and phase displayed no relationship with dCA.

Ageing is a risk factor for cerebrovascular disease and complications. A number of cerebral haemodynamic parameters change with age, including reductions in CBF volume and CBFv (Krejza et al. 1999; Ainslie et al. 2008; Lu et al. 2011). Yet, our current data show that the intrinsic ability of cerebral vessels to maintain stable flow in response to acute changes in BP is unaffected by ageing across the lifespan up to the age of 70 years. This suggests that the age-related decline in CBFv is not merely a result of impaired dCA. The ability of the cerebrovasculature to buffer transient changes in BP represents a vital defence mechanism protecting the brain from hypo- and hyperfusion (Claassen and Zhang 2011). Our data are in agreement with the previous studies, with smaller sample sizes or age group comparisons, which identified no reduction in dCA with older age using both squat-stand manoeuvres (Xing et al. 2017; Smirl et al. 2014; Oudegeest-Sander et al. 2014) or other dCA techniques (Yam et al. 2005; Carey et al. 2000; Dineen et al. 2011). We also show that dCA is not different between sexes when age is considered. Whilst some previous work has identified interactions between sex and dCA (Deegan et al. 2011; Labrecque et al. 2019a), suggesting this as possible explanation for increased orthostatic hypotension-related complications, the data from our large sample study did not show any interactions between sex across age ranges.

Another novel aspect of our study was that we examined the impact of the presence of CVD risk factors on the decline in dCA. Central obesity, hypertension, hypercholesterolemia, and T2DM represent major risk factors in the development of systemic vascular disease and complications (Seven 2015) including significantly increased risk of cerebrovascular disease (Law et al. 2009; Kivipelto et al. 2005; Pinto et al. 2004). Each risk factor individually or collectively is associated with endothelial dysfunction, increased arterial stiffness, alongside a range of other vascular abnormalities (Stapleton et al. 2008). Despite these vascular changes, none of these CVD risk factors included within our study were associated with a reduction in dCA when age in considered. Our group has previously shown that in a small sample of individuals with increased CVD risk, dCA is not different to that of young healthy individuals (Carter et al. 2020); with the current study, we confirm the original observation using a markedly larger sample size. To date, no other studies have assessed dCA using squat-stand manoeuvres in a population with these specific risk factors for CVD. Comparisons between previous studies that have assessed cerebral autoregulation in similar cohorts are challenging because of methodological differences. Previous studies employing squat-stand maneuverers and TFA examined one CVD risk factor, i.e., hypertension (Lipsitz et al. 2000; Eames et al. 2003) and T2DM (Huq et al. 2012) and also observed no change in dCA. Studies utilising the exact same dCA methods used in our study have observed no difference in patients with chronic obstructive lung disease (Lewis et al. 2019), in early stage Alzheimer’s (Claassen et al. 2009a), or even in heart transplant recipient patients (Smirl et al. 2014). Collectively, our data suggest that despite the vascular maladaptations that are associated with CVD risk factors, the intrinsic ability of the cerebral blood vessels to maintain stable flow upon fluctuations in BP is persevered.

Elevated cardio-respiratory fitness is associated with increased resting CBFv values (Ainslie et al. 2008) and enhanced cerebrovascular reactivity (Bailey et al. 2013), but its association with dCA is less clear. In fact, cardio-respiratory fitness may be important when assessing dCA (Labrecque et al. 2019b). Using a relatively large sample size, of moderately fit individuals, we found *V*O_2max_ is not related to variations in dCA. Interestingly, two previous studies concluded that higher *V*O_2max_ was related to attenuated dCA (Labrecque et al. 2017; Lind-Holst et al. 2011), whereas Aengevaeren et al. (2013) identified no effect of *V*O_2max_ on dCA. Disparities in the study findings are likely due to differences in dCA assessment methods, but could also be explained by including individuals with fitness levels at the lower and higher ends of the continuum. Moreover, differences in specific training status may alter dCA responses independent of *V*O_2max_; for example, the work by Labrecque et al. (2017) recruited individuals with a training load of 12 h per week for a minimum of 2 years, whereas in our study, we did not take into consideration training load/status but rather just cardio-respiratory fitness based on a maximum capacity exercise test. Whether any changes associated with improved/reduction in dCA are directly related to cardio-respiratory fitness or vascular/neural adaptations to chronic exercise requires further investigation. Our data provide some evidence, in a demographically varied cohort, using a single method of dCA assessment with TFA, suggesting that *V*O_2max_ has little impact on dCA, albeit within a small range of moderately fit individuals.

Our data further support a wealth of research that shows cBRS declines with age (Monahan 2007; Xing et al. 2017; Smirl et al. 2014; O'Mahony et al. 2000; La Rovere et al. 2008). We provide evidence that CVD risk factors are linked to reduced cBRS (Skrapari et al. 2007; Sakamoto et al. 2019; Madden et al. 2010) and cBRS across a broad age range in females is reduced in post-menopausal women compared to pre-menopausal (Barnes et al. 2012). We provide some evidence that this is could be explained by age, rather than the menopause accelerating any decline in cBRS. We acknowledge that further investigation is warranted to explore the impact of the menopause. The direct relationship between dCA and cBRS is complex. Understanding whether enhanced BP control leads to better control of CBF or vice visa is important in understanding how these regulatory mechanisms operate, and whether they should be the focus of interventions (Favre and Serrador 2019).

Our study provides evidence that cBRS parameters show no relationship with dCA normalised gain and dCA phase during forced BP oscillations, but do appear to have a relationship with dCA (absolute) gain. Absolute gain reflects absolute CBFv changes (Claassen et al. 2016), and thus with both CBFv and cBRS reducing with age, it is likely to result in a significant association between the two parameters, but when dCA gain is normalised for changes in BP no relationship is present. Interestingly, one previous study using TCD to measure rate of regulation and autoregulation index for dCA and the modified Oxford technique to estimate BRS, reported an inverse relationship between the two processes (Tzeng et al. 2010). This implies that the lower an individual’s BRS (i.e., reduced BP control), the more effective their dCA is at counteracting large fluctuations in BP and could imply an increased efficiency of dCA in protecting against the age-related decline in cBRS and various haemodynamic changes. On the other hand, previous studies utilising the same methods adopted in this present study concluded no relationship between dCA and cBRS parameters (Smirl et al. 2014; Aengevaeren et al. 2013). Therefore, the data from our study outline that despite having a significantly greater BP control at a younger age, this does not alter how well the cerebral vessels regulate blood flow during BP challenges. Differences in study findings may simply be explained the assessment of cBRS and dCA, as work by Horsman et al. (2014) demonstrated that in squat-stand manoeuvres at 0.10 Hz hysteresis is present which was not with the modified oxford technique. The squat-stand manoeuvres produce significant fluctuations in central blood volume and total peripheral resistance The overall input of the baroreceptors and neural feedback on dCA is complex and not well understood (Ainslie and Brassard 2014) with evidence from animal studies, showing that isolated dual elimination of baroceptor and chemoreceptor completely abolished cerebral autoregulation in dogs, whereas cerebral autoregulation was preserved in in sympathetically and parasympathetically denervated animals (Sagawa and Guyton 1961; Busija and Heistad 1984).

Our study utilises a large sample size, in a demographically varied cohort, employing the same technique and adhering to published guidelines. However, we acknowledge a number of limitations. First, the use of TCD assesses blood flow velocity rather than blood flow as arterial diameter is not taken into consideration and therefore a stable diameter cannot be verified. MCA diameter has been shown to be consistent during modest changes in CO_2_ (± 5 mmHg) (Ainslie and Hoiland 2014), as well as acute moderate changes in BP (Giller et al. 1993; Serrador et al. 2000); thus, our data should be interpreted with some caution. Second, we employed 0.10 Hz squat-stand manoeuvres only to interrogate dCA, incorporating 0.05 Hz manoeuvres and spontaneous oscillations may have provided an additional level of detail and strengthened interpretability of the results. Third, ageing was used as an individual variable within our analysis rather than incorporating it into the CVD risk factors section, as age does represent a major non-modifiable risk factor for CVD. Finally, the Bruce protocol was utilised for the assessment of *V*O_2max_ and using this protocol may have resulted in an underestimation of the *V*O_2max_ in the younger individuals.

In conclusion, we show that older age was associated with a decreased baroreflex sensitivity, but dCA appears to remain stable with ageing, sex, CVD risk, and cardio-respiratory fitness have little effect. Therefore, cerebral vessels regulate blood flow during acute BP challenges across the age span.

## Data Availability

The data that support the findings of this study are available from the corresponding author upon a reasonable request.
